# Discogenic cell transplantation directly from a cryopreserved state in an induced intervertebral disc degeneration canine model

**DOI:** 10.1002/jsp2.1013

**Published:** 2018-05-11

**Authors:** Syunsuke Hiraishi, Jordy Schol, Daisuke Sakai, Tadashi Nukaga, Isaac Erickson, Lara Silverman, Kevin Foley, Masahiko Watanabe

**Affiliations:** ^1^ Department of Orthopaedic Surgery, Surgical Science Tokai University School of Medicine Isehara Japan; ^2^ DiscGenics Inc. Salt Lake City Utah; ^3^ Semmes‐Murphey Clinic & Department of Neurosurgery University of Tennessee Health Science Center Memphis Tennessee

**Keywords:** cell transplantation, cryopreservation, degeneration, intervertebral disc, preclinical

## Abstract

A multitude of studies has indicated the potential of cell therapy as a method for intervertebral disc (IVD) regeneration. Transplantation of a variety of cells has been assessed and shown capable of deterring the rate of degeneration in animal models and in human clinical trials. In this study, a novel approach using human discogenic nucleus pulposus cells directly from their cryopreserved state was assessed. In an established canine disc degeneration model, the degeneration process was evaluated in IVDs receiving precultured discogenic cells, thawed‐only discogenic cells, and a saline sham injection after induction of degeneration. Degeneration progression was followed over time by the evaluation of the disc height index (DHI). Finally, after 12 weeks, the manipulated and control discs were explanted, histologically stained, and scored. Treated discs demonstrated retained DHI values for all treatment options. Histologic evaluations demonstrated significant improvement of matrix features compared to the sham. Moreover, thawed‐only cells function at least as well as precultured discogenic cells. In short, cell transplantation of human discogenic cells directly from their cryopreserved state can arrest disc height degeneration and maintain histological matrix features in a canine disc degeneration model. The presented work demonstrates the potential of an off‐the‐shelf cell therapy product to treat degenerative disc disease.

## 1 INTRODUCTION

Low back and neck pain are the largest causes of disability worldwide and constitute a crucial global health issue. Approximately 5% to 10% of the estimated 632 million low back pain patients globally will advance to a chronic condition.^1^, ^2^ Together, these compelling numbers engender a critical social–economic burden on society. For example, within the USA, 100 billion USD is spent annually on low back pain associated costs.^3^, ^4^ Currently, treatment options are limited and fail to restore or halt further advancement of the underlying pathology.^5^ Degeneration of the intervertebral disc (IVD) is widely considered to be a predominant cause of low back pain. IVD degeneration is hallmarked by a dysregulation in extracellular matrix (ECM) homeostasis.^6^, ^7^ The exact origin of IVD degeneration remains to be elucidated; however, the nucleus pulposus (NP) is believed to be the place of onset.^8^, ^9^ Progression of IVD degeneration is characterized by a decline in proteoglycan production, an increase in matrix degenerative proteins, and a switch from type II collagen to type I collagen production.^6^, ^7^ Moreover, NP cells undergo senescence and dedifferentiation toward a more fibrotic phenotype.^10^ The NP disorganization and height loss causes incorrect loading of adhering tendons promoting reorganization of tendon ECM to thicker and stiffer structures, further disrupting the mechanical features along the spine.^11^

Despite the well‐established pathway of the degeneration pathology, a curative treatment remains nonexistent. One hallmark of IVD degeneration that offers a promising therapeutic approach is the decrease in progenitor cell populations^12^ and overall NP cell populations.^10^, ^12^, ^13^ Promising developments in cell transplantations offer opportunities to replenish the degenerating IVD with new and active cell populations. Different cell types have been explored as a source for IVD regeneration (reviewed in Sakai and Schol^14^), including mesenchymal stem cells (MSCs),^15^, ^16^ chondrocytes,^17^ and IVD‐derived cells.^18^, ^19^ These in‐human studies showed positive effects with the introduction of new cell populations, by halting or limiting the degenerative process. However, selecting the right cell source is crucial for producing optimal results, as was exhibited by the first in‐human IVD cell therapy study.^20^ In this study of Haufe and Mork, hematopoietic stem cells were injected into the IVD of 10 patients, which led to no observed improvement in pain reporting or radiographic findings 1‐year posttransplantation.^20^ The largely avascular nature of the IVD gives the NP microenvironment an acidic, hypoglycaemic, hyperosmolar, and hypoxic disposition. This exceptional habitat poses an obstacle for the implanted cells to survive and actively contribute to *de novo* ECM generation. To circumvent these issues, investigations have been assessing the application of NP‐derived ‘discogenic’ cells, as a cell source for IVD regenerative therapy. Nevertheless, work from Hegewald et al. suggested a limited potency of NP cells derived from herniated IVD tissues.^21^ Therefore, investigators explored methods for enrichment and stimulation of active NP cells. Nukaga et al. applied a MSC coculture setup to reactivate NP cells and subsequently inserted them in an induced canine IVD degeneration model, resulting in improved histological and radiographic features.^22^ Liu et al. applied genetically modified NP cells, designed to overexpress connective tissue growth factor or tissue inhibitor of metalloproteinases which after transplantation resulted in maintained disc features and increased expression of ECM components.^23^ The first in‐human assessment by Meisel et al. applied autologous NP cells to limit degeneration onset from disc herniation treated by discectomy, which resulted in improvement in low back pain and disc hydration, contradicting the result from Hegewald et al.^18^, ^21^, ^24^ In a later in‐human trial by Mochida et al., applied transplantation of autologous NP cells reactivated by MSC coculture in IVDs adjacent to a fused IVD resulted in a lack of degeneration progression in the treated discs.^19^ Overall, these studies demonstrate the safety and potential of the transplantation of IVD‐derived cells as a treatment of IVD degeneration.

In order to enhance clinical translatability and reduce overall costs of a potential cell therapy, the product would ideally comprise an off‐the‐shelf (OTS) treatment. It has been shown that discogenic cells can be reactivated postcryopreservation;^22^, ^25^ however, this would still require additional culture of the discogenic cells prior to application. Here, we describe a novel OTS strategy for the transplantation of discogenic cells.^26^ The cells derived from human NP tissue were cultured for 4 to 6 weeks, and then frozen for long‐term storage in the vapor‐phase of liquid nitrogen. This study aims to assess the applicability of transplanting human discogenic cells directly from their cryopreserved storage condition as a treatment for IVD degeneration. In a canine disc degeneration model, safety and efficacy of this OTS approach were assessed and compared to the treatment of identical discogenic cells precultured for 2 weeks prior to delivery. Radiographic and histological images were used to assess their regenerative capacity.

## 2 MATERIALS AND METHODS

### 2.1 Disc degeneration model

This study was conducted in accordance with protocols approved by the Tokai University School of Medicine committee for safe animal experimentation (162031). Ten female chondrodystrophic beagle dogs (Kitayama Lab Co. Ltd., Nagano, Japan) were obtained at 12 months of age with an average weight of 8.5 ± 0.46 kg. Canine subjects were all in good conditions and plain X‐ray imaging confirmed the absence of lumbar disease. By intramuscular injection of 0.4 mg/kg midazolam (Astellas Pharma Inc., Tokyo, Japan), 0.02 mg/kg medetomidine (Kyoritsu Seiyaka Corp., Tokyo, Japan), and 0.4 mg/kg butorphanol tartrate (Meiji Seika Pharma Inc., Tokyo, Japan), canine subjects were rapidly sedated followed by continuous anesthetization of 2.5% isoflurane inhalation (Pfizer, New York City, New York, USA). Lumbar discs L5/6, L4/5, and L3/4 were exposed using a left anterior retroperitoneal approach. Under a fluoroscopy‐guided approach, an 18‐gauge needle with stopper was inserted through the annulus fibrosus (AF) into the NP. Through suction with a 10‐mL syringe, approximately 25 mg wet weight NP tissue was aspirated. The induced degeneration was allowed to progress for 2 weeks prior to treatment.^27^

### 2.2 Cell culture

Human adult IVD tissue was procured from a single organ donor (26‐year‐old male, BMI 22.9, nonsmoker) after consent, under supervision of an ethics review board. The tissue was transported in HypoThermosol (Biolife Solutions, Bothell, Washington, USA) with gentamycin and amphotericin B (Mediatech, Manassas, Virginia, USA). Once in the lab, the tissue was further dissected to be free of surrounding endplate material and washed to remove blood and other contaminants. Then, the cells isolated from the tissue were expanded over 4 to 6 weeks, causing changes that result in the phenotype of discogenic cells (US9487753‐B2).^26^ Finally, the cells were mixed with Profreeze‐CDM cryopreservation medium (Lonza, Basel, Switzerland) at the second passage, with 7.5% BloodStor dimethyl sulfoxide (DMSO) (Biolife Solutions) at low (1.5 × 10^6^) or high (15 × 10^6^) cell densities in cryovials, frozen using a controlled rate freezer and stored in the vapor phase of liquid nitrogen until further use.

To preculture the cells, the cryovials containing discogenic cells were opened inside a laminar flow cabinet, thawed, and suspended in Dulbecco’s modified Eagles medium nutrient mixture F‐12 Ham medium^19^ (DMEM/F12; Thermo Fisher, Waltham, Massachusetts, USA) containing 10% foetal bovine serum (FBS) and 50 μg/mL l‐ascorbic acid phosphate magnesium salt *n*‐hydrate. Discogenic cells were seeded at a density of 5.5 × 10^2^ cells/cm on 10 cm plate and cultured up to 70% to 80% confluency. During the expansion period, no passaging was required. Subsequently, the cells were cultured under a controlled 2% O_2_, 5% CO_2_, 37°C environment. After 2 weeks, discogenic cells were detached from their culture plates by incubation in 1 mL of 0.25% (wt/vol) trypsin/ethylenediaminetetraacetic acid (EDTA) (ThermoFisher) for 3 to 5 minutes, collected in 10% FBS DMEM/F12, spun‐down, counted by trypan blue exclusion method, and further diluted to cell concentrations of 1.5 × 10^6^ cells/mL or 15 × 10^6^ cell/mL.

### 2.3 Cell transplantation

Two weeks following NP aspiration, canine recipients were rapidly sedated as previously described. Canine subjects were positioned in a left lateral decubitus position under general 2.5% isoflurane inhalation anesthesia. Canines were randomly divided into a low‐dosage recipient or high‐dosage recipient groups. For each dog, 100 μL of cryopreserved OTS discogenic cells with 1.5 × 10^6^ cells/mL (OTS‐LOW, *n* = 5) or 15 × 10^6^ cells/mL (OTS‐HIGH, *n* = 5) were rapidly and completely defrosted using the ThawSTAR (MedCision, San Rafael, California, USA), after which the thawed cell suspension was mixed with 50 μL of 30 mg/mL sodium hyaluronate (HA) solution (molecular weight 650‐900 kDa, 1% HA; Lifecore Biomedical, Chaska, Minnesota, USA) via a double leur lock syringe connector by aspirating approximately 20 times back and forth. Concurrently, 100 μL 1.5 × 10^6^ cells/mL for low‐dose recipients of previously cultured cells (CUL‐LOW, *n* = 5) or 100 μL of 15 × 10^6^ cells/mL for high‐dose recipients of previously cultured cells (CUL‐HIGH, *n* = 5) were aliquoted and were identical to the OTS conditions, encapsulated in HA. Using a percutaneous fluoroscopy‐guided anterolateral approach, a 23‐gauge needle with stopper was inserted into the NP of lumbar discs. With a 0.5 mL glass syringe (Hamilton; Sigma‐Aldrich, St. Louis, Missouri, USA), both 100 μL of OTS or CUL suspensions were separately administered into L5/6, L4/5, or L3/4 in a randomly assigned order. Each dog also received 100 μL of sterile Dulbecco’s phosphate‐buffered saline (PBS) without calcium and magnesium (Thermo Fisher) injection (Sham, *n* = 10) in the remaining degenerated IVD. L6/7 functioned as a nonmanipulated healthy control throughout the study (*n* = 10). The cell transplantation was followed by 2 weeks of intramuscular injection of 0.15 mg/kg tacrolimus hydrate (Astellas Pharma Inc., Japan), an immune suppressant, approximately 5 days a week.

### 2.4 Hematology screening

Prior to NP aspiration and 4 weeks after cell transplantation, approximately 6 mL of blood was obtained and evaluated by the Health Science Research Institute, Inc. (Yokohama, Japan). A hematology, coagulation, and clinical chemistry assessment was performed for each dog.

### 2.5 Radiological assessment

**Figure 1:**
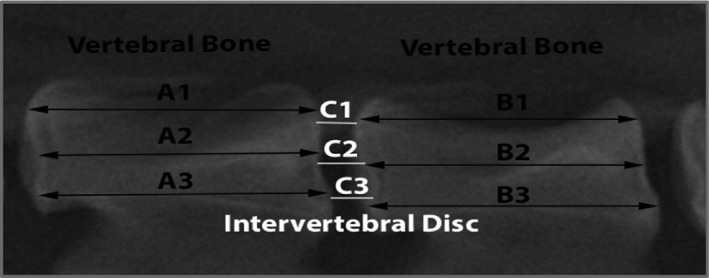
DHI assessment. A visual depiction of disc height index (DHI) assessment. The length on 3 separate locations is measured for the adjacent vertebrae and the intervertebral discs (IVDs). DHI is thereafter calculated via 2 × ((*C*1 + *C*2 + *C*3)/(*B*1 + *B*2 + *B*3 + *A*1 + *A*2 + *A*3)) × 100%

Plain lumbar X‐ray images were obtained from all canines 2 weeks prior, during, and 4, 8, and 12 weeks after cell transplantation. Images were taken by fluoroscopic imaging intensifier DHF‐105CX (Hitachi, Tokyo, Japan) with 80 kV, 2 mA at a distance of 100 cm. Images were uploaded in OsiriX‐lite (Pixmeo SARL, Bernex, Switzerland) and IVD height and vertebral height were converted to disc height index (DHI) (Figure 1) according to published protocols.^28^ In short, from a medial plane at 3 points, the height of involved IVD and neighboring vertebrae was determined. DHI was determined as 2 × ((*C*1 + *C*2 + *C*3)/(*B*1 + *B*2 + *B*3 + *A*1 + *A*2 + *A*3)) × 100%. Subsequently, changes in DHI for every assessed disc were calculated over time as the percentage of DHI relative to DHI prior to degeneration induction. Finally, DHI was calculated relative to the internal L6/7 reference control. All data were transferred to Microsoft Excel (Microsoft, Redmond, Washington, USA) and graphically presented by GraphPad Prism (GraphPad Inc., San Diego, California, USA).

### 2.6 Tissue explantation

Twelve weeks after cell transplantation, canines were rapidly sedated as previously described, followed by an excess intravenous injection of 300 mL 50 mg/mL P0776 pentobarbital sodium salt (Tokyo Chemical Industry Co., Tokyo, Japan). The lumbar sections of the spine were excised and both manipulated and control IVDs were macroscopically detached. Excess muscle, nerve, and fat tissue were manually removed. Tissue explants were transferred and stored at 4°C in 4% paraformaldehyde for 7 days, followed by 4°C decalcification solution A (Wako, Tokyo, Japan) for 7 days. Additionally, an array of organs was explanted from 2 randomly assigned dogs from both high‐ and low‐dose recipients and macroscopically examined for tumor formation or other apparent abnormalities (Table S1).

### 2.7 Histology

After fixation and decalcification, IVDs were cut through the center along their median plane. Macroscopic images were taken by an 8‐megapixel iSight camera with 1.5 μm pixel size (Apple, Cupertino, California, USA). IVDs were agitated, paraffinized, and sliced to 8 μm sections. Each explant was stained by hematoxylin/eosin (HE) and 1 g/L Safranin‐O (Merck, Kenilworth, New Jersey, USA) 800 mg/L Fast Green FCF staining (Merck) solution. Multiple images were captured by using a KEYENCE fluorescence microscope BZ‐9000 (KEYENCE, Osaka, Japan) and digitally merged via imaging stitching. Subsequently, all sections were independently scored by 3 researchers (H.S., J.S., and T.N.) according to an adapted version of the canine‐specific Bergknut scoring system, where Safranin‐O/Fast Green staining was used instead of alcian blue/Picrosirius Red staining to assess proteoglycan matrix abundance.^29^ Finally, tissue explants (Table S1) were similarly stained by HE to screen for histological abnormalities.

### 2.8 Immunohistochemistry

The presence of reminiscent human cells was evaluated by fluorescent immunohistochemistry staining targeting human leukocyte antigen (HLA) complexes of human discogenic cells. Sections were deparaffinized and incubated in 0.0005% proteinase (Sigma‐Aldrich, St. Louis, Missouri, USA) tris–HCl (pH 7.6) for 10 minutes at 37°C. Sections were rinsed in PBS and treated with 100 μL 3.0 IU/mL hyaluronidase type V (Sigma‐Aldrich), 0.05 IU/mL chondroitinase‐ABC (Sigma‐Aldrich) PBS for 60 minutes at 37°C. Subsequently, sections were rinsed and blocked with 3% bovine serum albumin (BSA), 0.2% Tween‐20 (Sigma‐Aldrich) PBS for 30 minutes at room temperature. Primary antibody antihuman HLA‐ABC Purified (BD BioScience, Frankilin Lakes, New Jersey, USA) was diluted 1:25 in 1% BSA, 0.2% Tween‐20 PBS, applied and incubated over night at 4°C. Subsequently, samples were rinsed, coated with secondary goat antimouse IgG/IgM Alexa Fluor 488 (Thermo Fisher), and incubated for 1 hour at room temperature. Sections were consecutively rinsed and mounted with VECTASHIELD HardSet Mounting Medium with 4′ 6‐diamidino‐2‐phenylindole (DAPI) (Vector Laboratories, Burligame, California, USA). Finally, all slides were analyzed by LSM 510 META confocal microscope (ZEISS, Oberkochen Germany).

### 2.9 Statistics

Significance for nontemporal assessments was determined by ordinary 1‐way analysis of variance (ANOVA) followed by Tukey’s multiple comparisons. Temporal assessments were determined by 2‐way ANOVA and corrected by Tukey’s multiple comparisons. Differences of *P* < .05 are considered statistically significant.

## 3 RESULTS

### 3.1 Disc degeneration model

Radiographic images, taken 2 weeks prior to transplantation, did not display any form of IVD associated diseases.

### 3.2 General findings

No direct injection‐related complications or surgical‐complications were observed, with the exception of 1 dog requiring additional stitching 1‐week posttransplantation due to the opening of the suture. The canines showed a slight decrease in average body weight 4‐week posttransplantation; however, body weight showed a nonrelevant increase throughout the rest of the study (Figure S1).

### 3.3 Hematology

Blood values, obtained 4 weeks after transplantation, indicated a statistically significant (*P* = .041) decrease in serum phosphorus levels (Table S2). No additional significant or clinically relevant changes were observed for the other serum values. White blood cell count showed a slight nonsignificant decrease, creatinine, bilirubin, urea, and ion levels remained stable.

### 3.4 IVD gross observations

**Figure 2:**
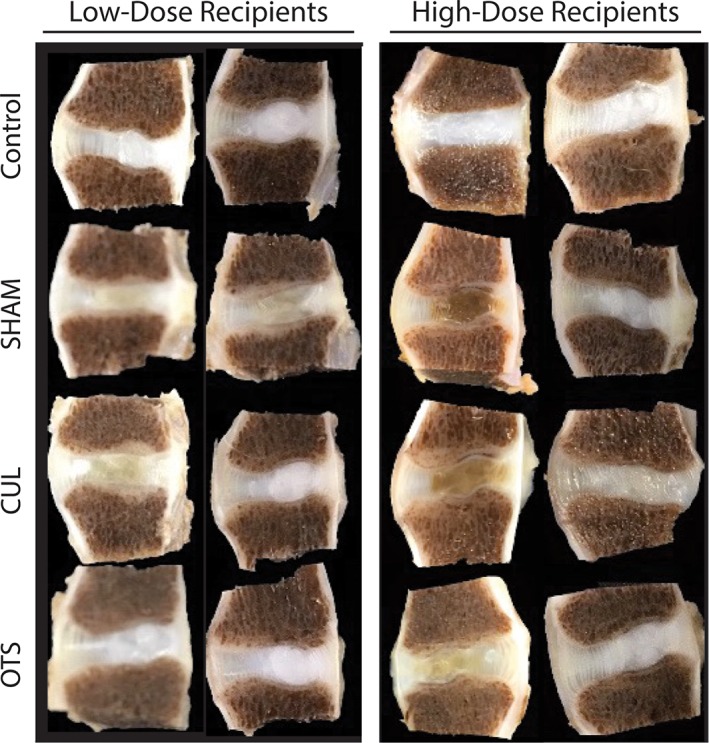
Macroscopic images of explanted intervertebral discs (IVDs). Macroscopic images of IVD explanted 12 weeks after cell transplantation, from high‐ and low‐dose recipients. Each column represents the complete IVD selection collected from 1 individual canine

After paraformaldehyde fixation and decalcification, nonmanipulated control discs maintained a white‐glossy appearance where the gel‐like NP structure remained distinguishable from the AF (Figure 2). Sham control samples lost their glossy appearance and the differentiation between NP and AF was diminished to completely lost. The CUL‐LOW treated disc resulted in discoloration of most discs, with 1 disc presenting apparent necrotic zones in the adjoining vertebrae (Figure S2). Moreover, a distinctive AF–NP border was diminished in 3 of the samples. CUL‐HIGH sections showed complete loss of a white‐glossy appearance in all but 1 section, with a mild to strongly diminished NP–AF border in all sections. OTS‐LOW did not show a strong discoloration in the NP area and the NP–AF border was well maintained in all but 1 sample. OTS‐HIGH, on the other hand, showed discoloration of the NP in 3 samples with a loss in NP–AF distinction in all but 2 samples.

### 3.5 Histology

**Figure 3:**
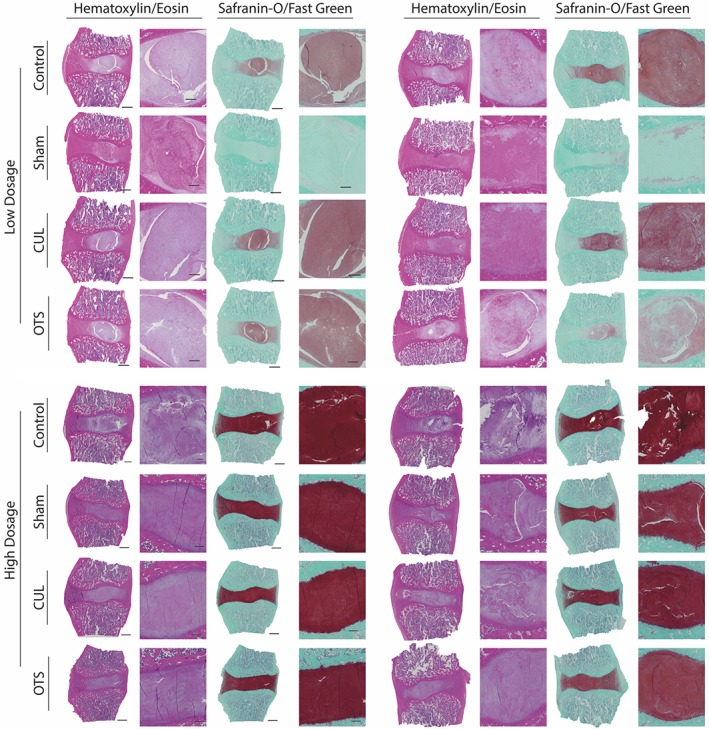
Histology overview. Selection of explanted intervertebral disc (IVD) sections stained by hematoxylin/eosin (left) and Safranin‐O/Fast Green (right) from a single dog per column. Sections are arranged in low‐dose (top) and high‐dose (bottom) recipients. Scale bar represent 1200 μm (left) and 300 μm (right; zoomed in)

**Figure 4:**
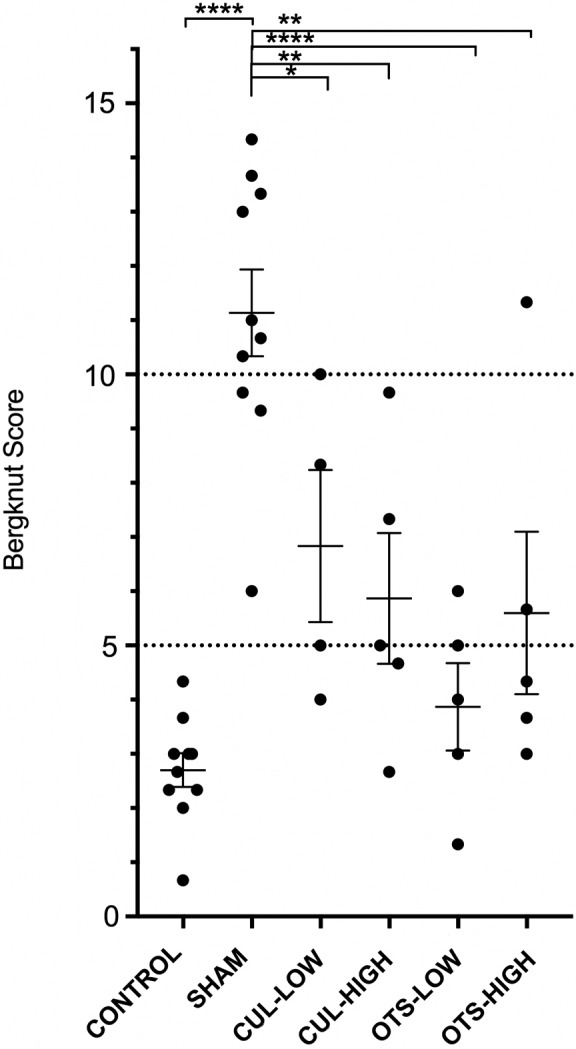
Histological classification. Scatter plot representing an overview of the average histological canine‐specific Bergknut score with standard error of the mean. Low and high indicates dosage of 1 × 10^5^ and 1 × 10^6^ cells per disc, respectively. Significance is determined by 1‐way analysis of variance (anova) followed by Tukey’s test and is signified as *P ≤ .05, **P ≤ .01, ***P ≤ .005, ****P ≤ .001

HE‐stained IVD sections confirmed the distinction of NP and AF in the L6/7 control discs as observed by macroscopic examination (Figure 3). Sham‐treated discs displayed a loss of AF–NP differentiation, ranging from a loss of a distinguishable border to a uniformly fibrous‐like structure. Treated discs presented mixed outcomes, ranging from an unaffected appearance to a complete transformation to a fibrotic structure. Similar to the macroscopic observation, the OTS‐LOW treated disc appears to maintain a distinctive NP core and layered AF structure, as observed by HE staining. Notably, however, the intensity of the Safranin‐O from the high‐dose group (include sham and control) was much more profound compared to the low‐dose group, despite concurrent staining of all samples. We hypothesize that these differences are due to different batches of fixation and decalcification reagents. Therefore, all comparisons were made relative to the internal control discs. OTS‐LOW Safranin‐O intensity was maintained in 3 out of 5 samples, while 1 sample showed slight intensity decrease, and 1 sample lacked Safranin‐O staining. OTS‐HIGH treated disc was able to maintain safranin‐O staining relatively to the control. However, strong fibrosis was observed in 1 of the samples. CUL‐LOW did show maintenance of Safranin‐O staining in all but 1 disc. Nevertheless, strong fibrotic tissue deposition was observed in 3 of the samples, including 1 disc presenting necrosis in the disc and far advanced fibrosis, as well as in the endplate and adjoining vertebrae. CUL‐HIGH also showed relative maintenance of Safranin‐O intensity; however, 2 samples showed ongoing fibrosis in the NP region. Treated samples did not reveal any infiltration of leukocytes or lymphocytes. Classification of the discs by the canine‐specific histological Bergknut scoring system revealed a strongly increased classification for the sham control compared to nonmanipulated controls (Figure 4) (increased score indicates more abnormal morphology). All treatment conditions resulted in significantly lower classifications compared to the sham, but not significantly different to the control. Additionally, an array of tissue biopsies (Table S1), including IVD biopsies, was explanted, which revealed no tumor formation or apparent abnormalities (data not shown).

### 3.6 Fluorescent immunohistochemistry

**Figure 5:**
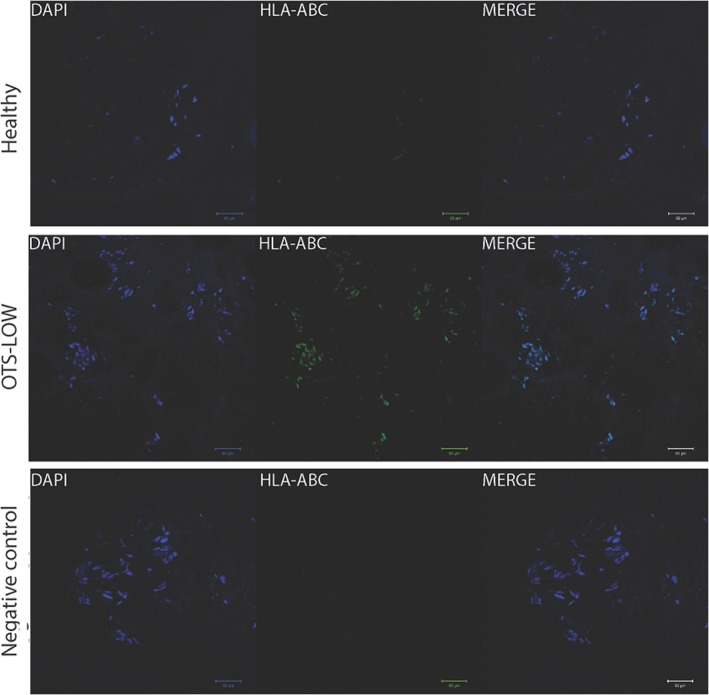
Human leukocyte antigen (HLA) targeted immunofluorescence staining. Explanted intervertebral disc (IVD) samples were stained by anti‐HLA‐ABC (green) and nuclei DAPI staining (blue). Nontreated (L6/7) and negative control (no primary antibody) showed no bright positive HLA‐ABC staining. Cryopreserved (off‐the‐shelf [OTS]‐LOW) cell transplantation treated disc showed clusters of HLA‐positive cells within the nucleus pulposus (NP). Scale bar represent 50 μm

HLA staining revealed occasional specific intensely stained cells within treated discs (Figure 5). DAPI and HLA‐positive cells were observed in the NP and sporadically observed in the inner AF of treated discs.

### 3.7 DHI assessment

**Figure 6:**
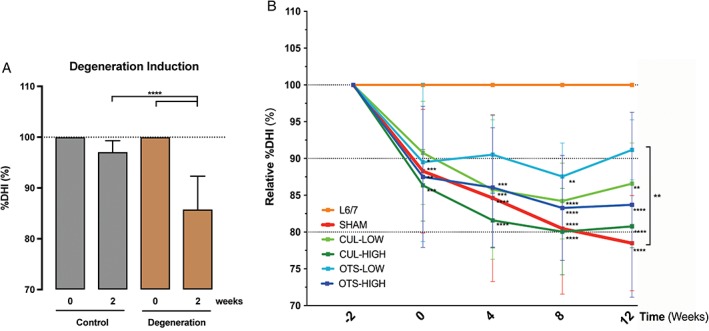
Disc height index (DHI) assessment. A, Average DHI determined for all discs prior and 2 weeks after degeneration induction. “Control” group represents nondegeneration L6/7 discs and “degeneration” are the collected L3/4, L4/5, and L5/6 DHI values. B, Average DHI tracked over 12 weeks. All values are set as a fraction of the DHI measurements prior to manipulation and subsequently placed relative to the internal control. Weeks represent the time after cell transplantation. Significance is determined by 2‐way analysis of variance (anova) and Tukey’s test. Significance is signified compared to the healthy control unless stated otherwise. *P ≤ .05, **P ≤ .01, ***P ≤ .005, ****P ≤ .001. Graph demonstrates mean values (±SD)

Following injury, radiographic images revealed a significant decrease in IVD height as signified by %DHI values (Figure 6). Two weeks after NP aspiration, the average DHI of aspirated discs was reduced by 14.2% ± 6.6%. The sham control displayed a continuous decrease in DHI, with a final reduction of 21.9% ± 6.2% at week 12. As expected, the nonmanipulated control group did not experience significant changes to DHI throughout the study. Transplantation of both CUL‐LOW and CUL‐HIGH showed a trend of diminished %DHI reduction compared to the sham control. OTS‐HIGH demonstrated more potent inhibition of %DHI reduction than the CUL groups. OTS‐LOW treatment had the best outcome, with a significantly (*P* = .0107) higher %DHI compared to the sham control, and showed a trend of slight DHI recovery.

## 4 DISCUSSION

The primary goal for this study was aimed at demonstrating the overall safety of the direct use of a cryopreserved discogenic cell transplantation product. Average body weight did show a slight decrease 4 weeks after transplantation; however, this was most likely a consequence of the tacrolimus hydrate administration, and remains within a healthy body weight range. An alteration noted in blood phosphorus levels was observed 4 weeks after transplantation; however, the values remained well within the healthy range of 2.8 to 7.7 mg/dL.^30^ White blood cell count did not show an increase despite the introduction of a xenogeneic cell population and limited 2‐week tacrolimus hydrate administration. Moreover, no observed immunogenic reactions were observed in situ, supporting the notion of a relative immune privileged environment within the IVD^31^ as well as of the cell population itself. The lack of rejection is similar to what was shown in an array of other studies utilizing xenogeneic stem and differentiated cell transplantations in the IVD, as reviewed in Sakai and Andersson,^32^ with beneficial outcomes, supporting the potential of an allogenic OTS treatment for IVD regeneration.

### 4.1 Disc height index

Disc height measurements revealed a successful induction of disc degeneration following NP aspiration. Previous investigations^22^, ^27^, ^33^ utilized the same canine model and found that transplantation of other cell types could limit or halt the rate of DHI reduction, but were unable to demonstrate restoration of the disc height. In contrast, the current study showed that one of the conditions, OTS‐LOW, displayed a trend of improved DHI and was statistically higher than sham control at week 12, indicating a trend of restoration of IVD feature by OTS‐LOW. CUL‐LOW, OTS‐HIGH, and CUL‐HIGH groups demonstrated a trend of plateauing around week 8, suggesting an arrest of degeneration progression.

### 4.2 Matrix preservation

Histological findings confirmed the trends observed for DHI measurements. Successful degeneration induction was confirmed by Bergknut classification by comparing the control to sham samples, demonstrating a significant difference of 8.4 points. In general, treatment with discogenic cells overall resulted in maintenance of the healthy NP characteristics. Of the treatment groups, CUL‐LOW treatment showed a large variation in histological outcomes and had on average a more abnormal appearance. One disc presented strong necrotic zones in adjacent vertebrae and a large fibrotic NP tissue deprived of Safranin‐O staining (Figure S2). These findings are likely a result of a surgical error during transplantation or introduced pathogens during cell preparation, and have therefore been removed from the histological examinations. In contrast, other treatments showed more reliable preserving ECM organization and scored an average lower; however, both OTS‐HIGH and CUL‐HIGH had 1 disc close to the sham average. On the other hand, OTS‐LOW showed a strong capacity in maintaining NP features as well as preserving safranin‐O staining. Interestingly, HLA‐positive cells were occasionally detected in both CUL and OTS treated discs within the NP, indicating that some percentage of the transplanted cells may have the capacity to survive within the degenerating IVD. Altogether, these results suggest that cells injected directly from a cryopreserved state are capable of halting disc degeneration.

### 4.3 OTS treatment

Cell transplantation has gained significant momentum and initial cell products have reached the clinics.^34^ A critical component for most cell transplantation products is the use of cryopreservation, and therefore, the effects of cryopreservation have been well studied.^35^ The effect of cryopreservation on MSCs^36^, ^37^ and hematopoietic stem cells,^38^, ^39^ related to the potential as cell therapy products, overall demonstrates a minimal effect on cell viability and bioactivity. For example, cryopreservation of adipose tissue‐derived MSCs did not result in any alterations in morphology, growth rate, karyotype, or marker expression. As well, there was no change in the capacity to react to stimuli or differentiation potential.^36^ Similar results are observed with hematopoietic stem cells.^40^ Moreover, previous work from Tanaka et al.^25^ demonstrated that cryopreserved NP cells possessed similar ECM production potential and proliferation rates after an in vitro coculture system compared to nonfrozen NP cells. Subsequently, Nukaga et al.^22^ showed that implantation of cryopreserved and reactivated NP cells resulted in similar regenerative effects in a canine model. Although both studies demonstrated that NP cells preserve their cell activity after cryopreservation, they both applied a reactivation coculture system to boost cell activity postcryopreservation. In the current study, cells were either transplanted directly from a frozen state or transplanted after 2 weeks of culture. Our OTS strategy outperformed identical cells transplanted after 2 weeks of preculturing, confirming that cryopreservation has limited effect on cell potency, similar to observations from previous studies.^22^, ^25^

The superiority of the OTS treatment is likely a consequence of a loss of potency of the precultured cells, which could result in changes to the original discogenic cell phenotype. A variety of studies have indicated a change of NP cell characteristics with extended culture, which might explain the enhanced outcomes for the OTS cells.^12^, ^41^, ^42^ In particular considering no stimulating factors were supplemented to the media. However, additional growth factor supplementation and additional culture time would significantly increase production costs and scheduling challenges between cell transplantations. Therefore, the direct application of cryopreserved, OTS discogenic cells poses a potentially promising and novel approach to the treatment of IVD degeneration.

Finally, the cell density of the transplantation has been shown to be an important factor for the outcome of the therapy.^33^ Serigano et al. revealed that either too many or too few cells can result in a limited regenerative outcome.^33^ Due to the avascular nature of the IVD, oxygen and nutrient accessibility is restricted. The introduction of additional cell populations could further increase the demand for nutrients, potentially limiting cell activity and survival for transplanted and endemic cells. In our study, both dosages resulted in a retarded DHI degeneration rate. Overall, the low‐dose transplantations appear most efficient in maintaining the DHI; however, for the histological observations, only a slight outperformance can be observed in the low dosage OTS strategy.

In short, discogenic cell transplantation did not result in any observed adverse systemic effects. Discogenic cell transplantation established a significant and clinically relevant improvement in histological IVD features and DHI, by halting the progression of degeneration or restoring the damaged matrix. The injection of low‐dose OTS discogenic cells demonstrated a significant and strong reduction in DHI loss and attained histological scores close to nondegenerating discs. Transplantation of directly thawed cells was superior to precultured cells in maintaining IVD characteristics. In conclusion, OTS discogenic cell transplantation appears to be a safe and effective treatment against induced chondrodystrophic canine disc degeneration, suggesting the use of cell transplantation populations directly from their cryopreserved state to be a viable transplantation strategy for human use.

## Supporting information


**Figure S1**. Body weight. Measurement of body weight prior to radiographic analysis overtime. Data are presented as the mean body weight relative to body weight at the time of cell transplantation. Error bars indicate SD.

**Figure S2**. Necrosis observed in CUL‐LOW treated disc. IVD treated with precultured discogenic cells at low cell density (CUL‐LOW) explanted 12 weeks posttransplantation revealed clear necrotic areas (as marked by arrow head) in the IVD and adjoining vertebrae as observed in macroscopic and histologic sections. Moreover, Safranin‐O staining was depleted and a clear fibrotic structure was formed at the site of the NP.

**Table S1**. Overview tissue biopsies.

**Table S2**. Overview Average Laboratory Values of Canine Blood Profiles.
