# Supplementary material for: Discogenic cell transplantation directly from a cryopreserved state in an induced intervertebral disc degeneration canine model
Source: JOR Spine. 2018 May 11;1(2):e1013. doi: 10.1002/jsp2.1013 (PMC6686803; doi:10.1002/jsp2.1013)
Supplement: Supplementary file 1 — Figure S1. Body weight. Measurement of body weight prior to radiographic analysis overtime. Data are presented as the mean body weight relative to body weight at the time of cell transplantation. Error bars indicate SD. [file JSP2-1-e1013-s001.docx]

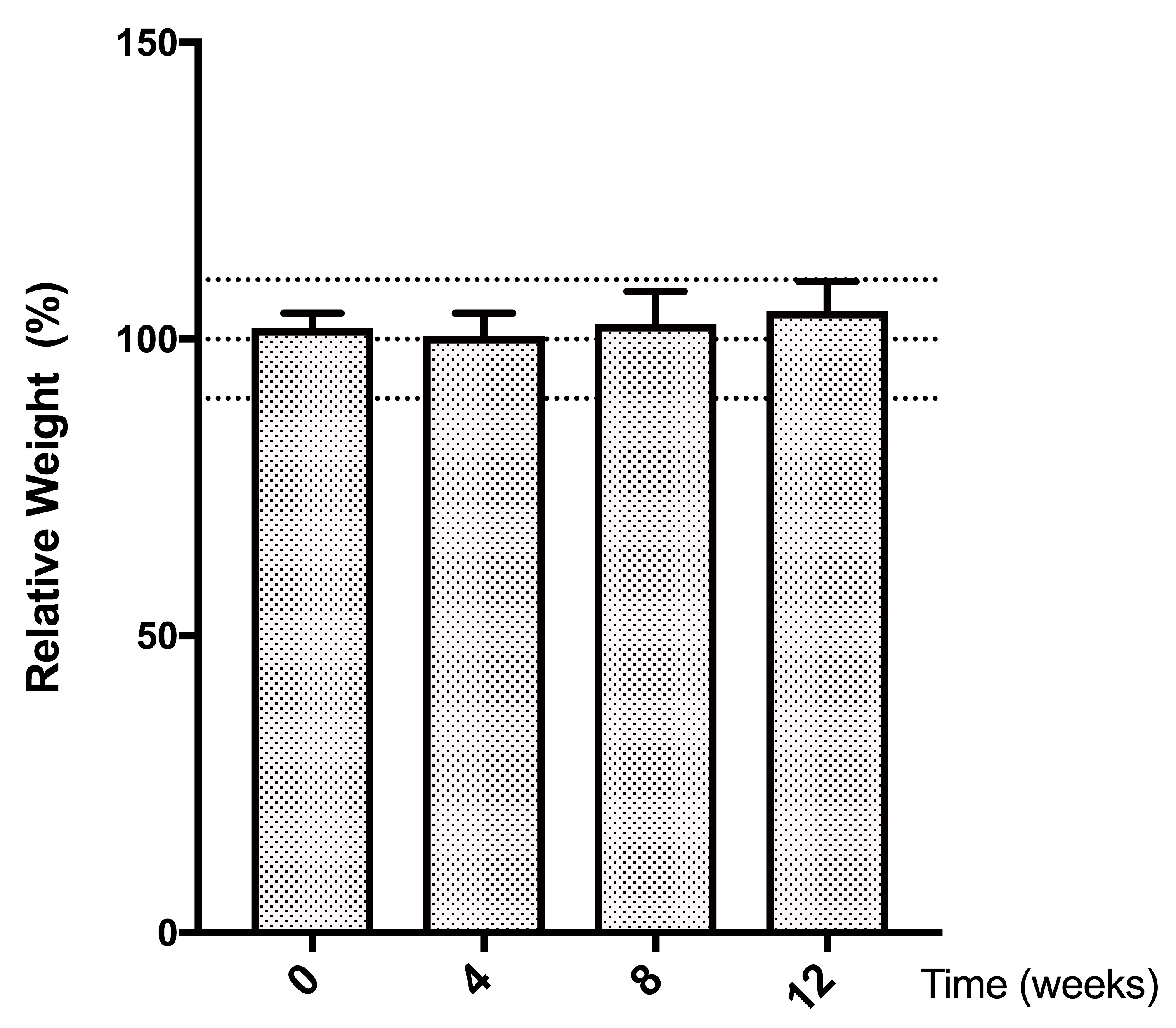


**Supplementary Figure 1.| Body Weight**

Measurement of body weight prior to radiographic analysis overtime. Data is presented as the mean body weight relative to body weight at time of cell transplantation. Error bars indicate standard deviation.
