# Supplementary material for: Discogenic cell transplantation directly from a cryopreserved state in an induced intervertebral disc degeneration canine model
Source: JOR Spine. 2018 May 11;1(2):e1013. doi: 10.1002/jsp2.1013 (PMC6686803; doi:10.1002/jsp2.1013)
Supplement: Supplementary file 2 — Figure S2. Necrosis observed in CUL‐LOW treated disc. IVD treated with precultured discogenic cells at low cell density (CUL‐LOW) explanted 12 weeks posttransplantation revealed clear necrotic areas (as marked by arrow head) in the IVD and adjoining vertebrae as observed in macroscopic and histologic sections. Moreover, Safranin‐O staining was depleted and a clear fibrotic structure was formed at the site of the NP. [file JSP2-1-e1013-s002.docx]

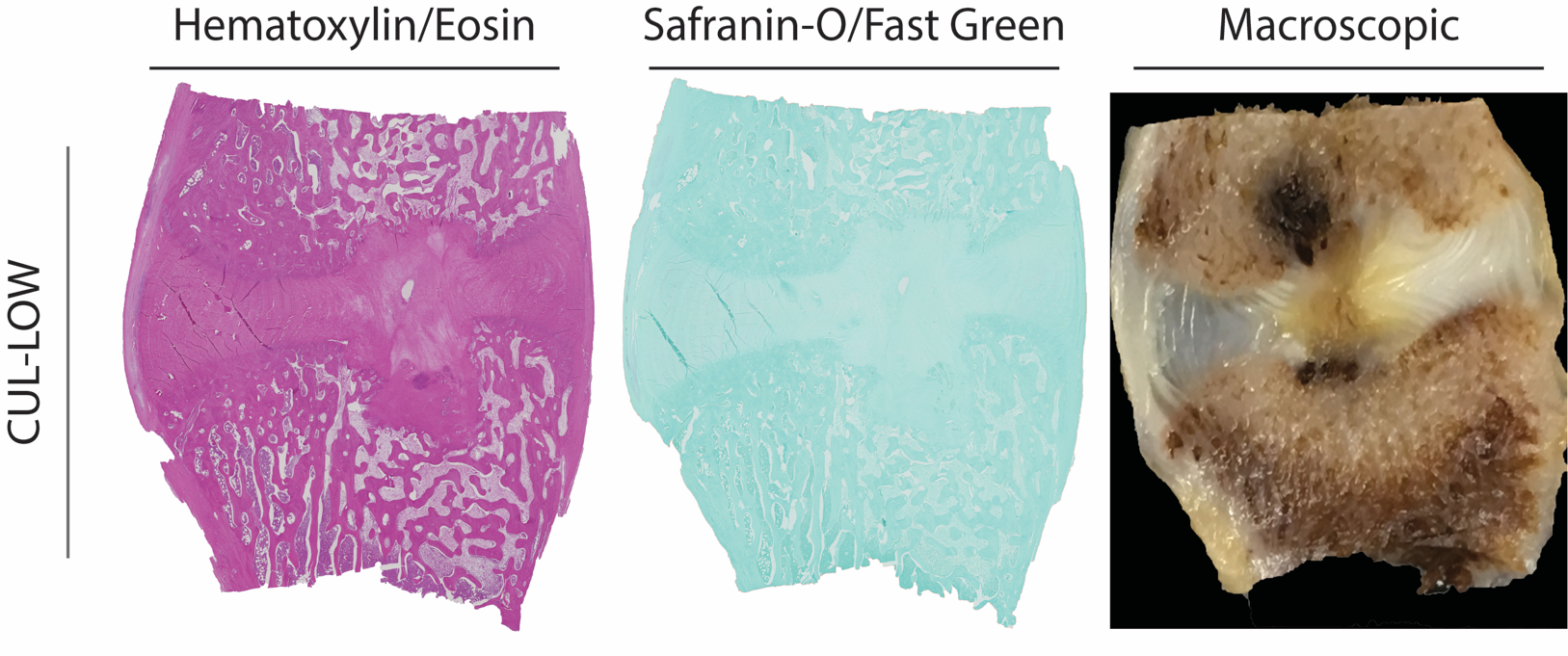


**Supplementary Figure 2.| Necrosis observed in CUL-LOW treated disc**

Intervertebral disc treated with pre-cultured discogenic cells at low cell density (CUL-LOW) explanted 12 weeks post transplantation revealed clear necrotic areas (as marked by arrow head) in the IVD and adjoining vertebrae, as observed in macroscopic and histologic sections. Moreover, Safranin-O staining was depleted and a clear fibrotic structure was formed at the site of the nucleus pulposus.
