# Supplementary material for: Discogenic cell transplantation directly from a cryopreserved state in an induced intervertebral disc degeneration canine model
Source: JOR Spine. 2018 May 11;1(2):e1013. doi: 10.1002/jsp2.1013 (PMC6686803; doi:10.1002/jsp2.1013)
Supplement: Supplementary file 3 — Table S1. Overview tissue biopsies. [file JSP2-1-e1013-s003.docx]

| **Tissue Biopsy** |
| --- |
| Skin |
| Mammary gland |
| Lymph node |
| Salivary gland |
| Sternum Bone |
| Sternum Bone Marrow |
| Femur Bone |
| Femur Bone Marrow |
| Thymus |
| Trachea |
| Bronchi |
| Lung |
| Heart |
| Thyroid |
| Parathyroid |
| Tongue |
| Esophagus |
| Stomach |
| Duodenum |
| Small Intestine |
| Large Intestine |
| Liver |
| Gall bladder |
| Pancreas |
| Spleen |
| Kidney |
| Adrenal Gland |
| Urinary Bladder |
| Seminal Vesicle |
| Ovary |
| Uterus |
| Vagina wall |
| Brain section |
| Pituitary gland |
| Spinal cord |
| Eye |
| Eye Adnexa |

**Supplementary Table 1.| Overview tissue biopsies**

An overview of all tissues explanted for examination of tumor formation or other apparent abnormalities.
