# Supplementary material for: Discogenic cell transplantation directly from a cryopreserved state in an induced intervertebral disc degeneration canine model
Source: JOR Spine. 2018 May 11;1(2):e1013. doi: 10.1002/jsp2.1013 (PMC6686803; doi:10.1002/jsp2.1013)
Supplement: Supplementary file 4 — Table S2. Overview Average Laboratory Values of Canine Blood Profiles. [file JSP2-1-e1013-s004.docx]

|  |  | Baseline | | | Week 4 | | |  | *p* |
| --- | --- | --- | --- | --- | --- | --- | --- | --- | --- |
| Total Protein | mg/dL | 6.3 | ± | 0.4 | 6.3 | ± | 0.4 |  | >0.9999 |
| Albumin | mg/dL | 2.8 | ± | 0.3 | 2.8 | ± | 0.1 |  | >0.9999 |
| Urea Nitrogen | mg/dL | 13.9 | ± | 1.9 | 13.9 | ± | 2.3 |  | >0.9999 |
| Creatinine | mg/dL | 0.5 | ± | 0.1 | 0.6 | ± | 0.1 |  | 0.286 |
| Cholesterol | mg/dL | 123.2 | ± | 14.9 | 128.1 | ± | 25.1 |  | >0.9999 |
| Neutral fat | mg/dL | 22.9 | ± | 5.9 | 26.0 | ± | 11.0 |  | >0.9999 |
| Bilirubin | mg/dL | 0.1 | ± | 0.0 | 0.1 | ± | 0.0 |  | >0.999 |
| AST | U/L | 68.2 | ± | 36.4 | 42.6 | ± | 6.5 |  | 0.286 |
| ALT | U/L | 40.3 | ± | 9.3 | 35.7 | ± | 6.5 |  | 0.737 |
| ALP | U/L | 252.3 | ± | 110.4 | 206.6 | ± | 61.8 |  | 0.813 |
| γ－ＧＴ | U/L | 3.2 | ± | 1.5 | 4.3 | ± | 1.1 |  | 0.425 |
| Na | mEq/L | 146.7 | ± | 0.9 | 146.5 | ± | 1.4 |  | >0.9999 |
| K | mEq/L | 4.6 | ± | 0.4 | 4.5 | ± | 0.3 |  | >0.9999 |
| Cl | mEq/L | 112.1 | ± | 2.0 | 112.5 | ± | 2.0 |  | >0.9999 |
| CA | mg/dL | 10.3 | ± | 0.3 | 10.3 | ± | 0.2 |  | >0.9999 |
| P | mg/dL | 5.5 | ± | 0.7 | 4.6 | ± | 0.3 |  | 0.041 |
| GLU | mg/dL | 86.1 | ± | 9.9 | 77.0 | ± | 5.5 |  | 0.279 |
| WBC | (x10^3)/μL | 11.8 | ± | 3.0 | 10.7 | ± | 2.3 |  | 0.897 |
| RBC | (x10^6)/μL | 699.2 | ± | 44.4 | 711.7 | ± | 38.9 |  | >0.9999 |
| Hb | g/dL | 16.6 | ± | 1.2 | 16.5 | ± | 1.0 |  | >0.9999 |
| EVF | % | 46.4 | ± | 3.3 | 46.6 | ± | 2.1 |  | >0.9999 |
| MCV | fL | 66.4 | ± | 3.4 | 65.6 | ± | 3.6 |  | >0.9999 |
| MCH | pg | 23.7 | ± | 0.9 | 23.2 | ± | 0.6 |  | 0.730 |
| MHCH | % | 35.7 | ± | 1.8 | 35.5 | ± | 2.1 |  | >0.9999 |
| PLT | (x10^6)/μL | 22.6 | ± | 11.8 | 28.3 | ± | 5.8 |  | 0.730 |
| PT | s | 9.1 | ± | 0.6 | 9.3 | ± | 1.0 |  | >0.9999 |
| PT INR | s | 0.9 | ± | 0.1 | 0.9 | ± | 0.1 |  | >0.9999 |
| PT activity | s | 135.9 | ± | 8.0 | 129.2 | ± | 19.0 |  | >0.9999 |
| aPPT | s | 15.0 | ± | 0.0 | 15.0 | ± | 0.0 |  | >0.9999 |
|  |  |  |  |  |  |  |  |  |  |

## Supplementary Table 2.| Overview Average Laboratory Values of Canine Blood Profiles

Summarized overview of blood profiles of all 10 dogs, just prior to degeneration induction (baseline) and 4 weeks after cell therapy. P value determined by multiple t-test using the two-stage linear step-up procedure of Benjamini, Krieger and Yekutieli, with Q = 1%. (AST; Aspartate transaminase, ALT; Alanine transaminase, ALP; Alkaline phosphatase, γ-GT; Gamma-glutamyl transpeptidase, Na; Sodium, K; Potassium, Cl; Chloride, Ca; Calcium, P; Phosphor, GLU; Glucose, WBC; White blood cells, RBC; Red blood cells, Hb; Hemoglobin, EVF; Erythrocyte volume fraction, MCV; Mean corpuscular volume, MCH; Mean corpuscular hemoglobin, MCHC; Mean corpuscular hemoglobin concentration, PLT; Platelet, PT; Prothrombin time, PT INR; Prothrombin time according to international normalized ratio, aPPT; Activated partial thromboplastin time)
